# Philadelphia Dispensary

**Published:** 1839-02-23

**Authors:** 


### PHILADELPHIA DISPENSARY.

*Report of Cases treated from the* 1*st to the* 31*st of January, inclusive.*

| | | |
|---|---|---|
| Whole number - - - | | 195 |
| Discharged cured - - - | 130 | |
| Dead - - - - - - | 11 | —141 |
| Remaining - - - - | | 54 |

Amongst the deaths, there were from

| | |
|---|---|
| Phthisis - - - - - - - - | 5 |
| Pleuro-pneumonia - - - - - | 1 |
| Double pneumonia - - - - - | 2 |
| Pneumonia supervening on mania-a-potu - | 1 |
| Cynanche trachealis - - - - | 1 |
| Uncertain - - - - - - - | 1 |
| Total - - - - - - - | 11 |

